# Supplementary material for: Heparin-based hydrogel scaffolding alters the transcriptomic profile and increases the chemoresistance of MDA-MB-231 triple-negative breast cancer cells
Source: Biomater Sci. 2020 Feb 13;8(10):2786–96. doi: 10.1039/c9bm01481k (PMC7497406; doi:10.1039/c9bm01481k)
Supplement: Supplementary file 2 [file BM-008-C9BM01481K-s002.zip › Supplementary File 4/HP-B hydrogel + EGF (s)vControl/Pathways/my_analysis.Gsea.1545201170987/HALLMARK_NOTCH_SIGNALING.html]

Details for gene set HALLMARK\_NOTCH\_SIGNALING[GSEA]

|  || Dataset | expr.class.cls#HYDEGF\_versus\_CONTROL.class.cls#HYDEGF\_versus\_CONTROL\_repos |
| Phenotype | class.cls#HYDEGF\_versus\_CONTROL\_repos |
| Upregulated in class | CONTROL |
| GeneSet | HALLMARK\_NOTCH\_SIGNALING |
| Enrichment Score (ES) | -0.26735926 |
| Normalized Enrichment Score (NES) | -0.88684326 |
| Nominal p-value | 0.60324824 |
| FDR q-value | 0.7510123 |
| FWER p-Value | 1.0 |
Table: GSEA Results Summary

  

Fig 1: Enrichment plot: HALLMARK\_NOTCH\_SIGNALING      
 Profile of the Running ES Score & Positions of GeneSet Members on the Rank Ordered List

  

| PROBE | DESCRIPTION (from dataset) | GENE SYMBOL | GENE\_TITLE | RANK IN GENE LIST | RANK METRIC SCORE | RUNNING ES | CORE ENRICHMENT || 1 | FZD7 | na |  |  | 573 | 2.041 | 0.0520 | No |
| 2 | PRKCA | na |  |  | 865 | 1.773 | 0.1080 | No |
| 3 | CCND1 | na |  |  | 2684 | 1.084 | 0.0566 | No |
| 4 | KAT2A | na |  |  | 3035 | 0.996 | 0.0783 | No |
| 5 | DTX2 | na |  |  | 3356 | 0.932 | 0.0990 | No |
| 6 | PPARD | na |  |  | 3488 | 0.907 | 0.1285 | No |
| 7 | FZD5 | na |  |  | 4153 | 0.791 | 0.1256 | No |
| 8 | NOTCH1 | na |  |  | 4284 | 0.769 | 0.1497 | No |
| 9 | PSEN2 | na |  |  | 4592 | 0.722 | 0.1626 | No |
| 10 | CUL1 | na |  |  | 5551 | 0.570 | 0.1356 | No |
| 11 | HES1 | na |  |  | 5622 | 0.558 | 0.1543 | No |
| 12 | NOTCH2 | na |  |  | 7202 | 0.343 | 0.0857 | No |
| 13 | ST3GAL6 | na |  |  | 8352 | 0.202 | 0.0339 | No |
| 14 | LFNG | na |  |  | 8516 | 0.185 | 0.0328 | No |
| 15 | DTX4 | na |  |  | 12950 | -0.335 | -0.1850 | No |
| 16 | FBXW11 | na |  |  | 13627 | -0.430 | -0.2030 | No |
| 17 | APH1A | na |  |  | 13804 | -0.459 | -0.1938 | No |
| 18 | TCF7L2 | na |  |  | 15215 | -0.672 | -0.2404 | Yes |
| 19 | SKP1 | na |  |  | 15444 | -0.710 | -0.2238 | Yes |
| 20 | FZD1 | na |  |  | 15551 | -0.732 | -0.1999 | Yes |
| 21 | ARRB1 | na |  |  | 15570 | -0.737 | -0.1713 | Yes |
| 22 | RBX1 | na |  |  | 16070 | -0.838 | -0.1638 | Yes |
| 23 | JAG1 | na |  |  | 16098 | -0.843 | -0.1313 | Yes |
| 24 | MAML2 | na |  |  | 16996 | -1.089 | -0.1344 | Yes |
| 25 | SAP30 | na |  |  | 17712 | -1.340 | -0.1180 | Yes |
| 26 | PSENEN | na |  |  | 17853 | -1.415 | -0.0685 | Yes |
| 27 | WNT5A | na |  |  | 18187 | -1.597 | -0.0218 | Yes |
| 28 | NOTCH3 | na |  |  | 18525 | -1.857 | 0.0351 | Yes |
Table: GSEA details [plain text format]

  

Fig 2: HALLMARK\_NOTCH\_SIGNALING      
 Blue-Pink O' Gram in the Space of the Analyzed GeneSet

  

Fig 3: HALLMARK\_NOTCH\_SIGNALING: Random ES distribution      
 Gene set null distribution of ES for **HALLMARK\_NOTCH\_SIGNALING**
